# Self vs. Other Focus: Predicting Professionalism Remediation of Emergency Medicine Residents

**DOI:** 10.5811/westjem.2017.11.35242

**Published:** 2017-12-14

**Authors:** Robert E. Thaxton, Woodson S. Jones, Fred W. Hafferty, Carolyn W. April, Michael D. April

**Affiliations:** * San Antonio Uniformed Services Health Education Consortium, Department of Emergency Medicine, San Antonio, Texas; † San Antonio Uniformed Services Health Education Consortium, Department of Graduate Medical Education, San Antonio, Texas; ‡ University of Minnesota Medical School, Department of Behavioral Sciences, Minneapolis, Minnesota; § University of Texas Health Sciences Center San Antonio, Department of Medicine, San Antonio Texas

## Abstract

**Introduction:**

Unprofessionalism is a major reason for resident dismissal from training. Because of the high stakes involved, residents and educators alike would benefit from information predicting whether they might experience challenges related to this competency. Our objective was to correlate the outcome of professionalism-related remedial actions during residency with the predictor variable of resident response to a standardized interview question: “Why is Medicine important to you?”

**Methods:**

We conducted a professional development quality improvement (QI) initiative to improve resident education and mentorship by achieving a better understanding of each resident’s reasons for valuing a career in medicine. This initiative entailed an interview administered to each resident beginning emergency medicine training at San Antonio Military Medical Center during 2006–2013. The interviews uniformly began with the standardized question “Why is Medicine important to you?” The residency program director documented a free-text summary of each response to this question, the accuracy of which was confirmed by the resident. We analyzed the text of each resident’s response after a review of the QI data suggested an association between responses and professionalism actions (retrospective cohort design). Two associate investigators blinded to all interview data, remedial actions, and resident identities categorized each text response as either self-focused (e.g., “I enjoy the challenge”) or other-focused (e.g., “I enjoy helping patients”). Additional de-identified data collected included demographics, and expressed personal importance of politics and religion. The primary outcome was a Clinical Competency Committee professionalism remedial action.

**Results:**

Of 114 physicians starting residency during 2006–2013, 106 (93.0%) completed the interview. There was good inter-rater reliability in associate investigator categorization of resident responses as either self-focused or other-focused (kappa coefficient 0.85). Thirteen of 50 residents (26.0%) expressed self-focus versus three of 54 (5.4%) residents expressed other-focus experienced professionalism remedial actions (p<0.01). This association held in a logistic regression model controlling for measured confounders (p=0.02).

**Conclusion:**

Self-focused responses to the question “Why is Medicine important to you?” correlated with professionalism remedial actions during residency.

## INTRODUCTION

Wynia et al. define medical professionalism as a “normative belief system about how best to organize and deliver health care.”^1^ There is evidence to support that professionalism is critical to the competent practice of medicine. Papadakis et al. demonstrated associations between state medical board disciplinary actions and documentation of unprofessional behavior during medical school^2^,^3^ or residency.^4^

The literature reports myriad strategies to remediate unprofessional behavior. Examples include mental health assessments, professional mentorship, role-modeling, remediation assignments, and building social support networks.^5^,^6^ Despite these many options, residency program directors (PDs) consistently report doubts that their professionalism remediation efforts are effective.^7^–^9^ Hence, the existing literature highlights the challenges of a reactionary approach to professional development.

This paper investigates the relationship between resident lapses in professionalism with residents’ responses to the question: “Why is Medicine important to you?” We hypothesized that a greater proportion of residents providing self-focused responses would experience professionalism disciplinary actions compared to residents expressing other-focused values.

## METHODS

The study setting was an academic urban tertiary care military hospital. The study participants were emergency medicine (EM) residents, all of whom were active duty service members. All residents beginning training between July 2006 and July 2013 were eligible for study inclusion except for residents starting training in July 2012 (class of 2015), as the lead author was absent that month. Exclusion criteria included residents opting out of interview participation or unable to participate due to scheduling conflicts.

In July 2006, we started a professional development quality improvement (QI) initiative entailing a standardized interview administered to all residents beginning EM training. The aim was to facilitate faculty teaching of residents through ascertainment of resident professional values, or trans-situational and inherently desirable goals related to their pursuit of a career in medicine.^10^ Upon review of the QI data, we believed our findings to be of interest to the medical education community. We subsequently received approval from our institutional review board to analyze the interview data for research purposes (retrospective cohort design).

The PD administered all interviews during the initial month of residency before any educational or clinical evaluations. To maintain a non-threatening environment, he held all interviews in a private room without recordings. The interviews uniformly began with the standardized question: “Why is Medicine important to you?” The PD tailored subsequent interview questions to the individual resident to elaborate on their answers. For example, an initial resident response of “because I want to help people” might be followed by the question “why is helping people important to you?” Other interview questions underwent minor modifications during the study period (Appendix Table), but the first question remained unchanged.

**Population Health Research Capsule**

What do we already know about this issue?

Professionalism is an important clinical competency. State medical board disciplinary actions are associated with unprofessional behavior during training.

What was the research question?

Do resident professionalism remedial actions correlate with responses to the question: “Why is Medicine important to you?”

What was the major finding of the study?

The incidence of professionalism remedial actions was higher among residents with self-focused vs other-focused responses.

How does this improve population health?

This association may aid medical educators in identifying residents who may benefit from additional attention to professional development.

Following each interview response, the PD entered de-identified free-text summaries of the interview responses into a secure Excel database (version 14, Microsoft, Redmond, WA). Upon completion of all interview questions, he showed the text for all responses to the resident for confirmation of text accuracy. He maintained records only of the final text approved by each resident.

The independent variable defining the study cohorts comprised each resident’s first unambiguous response to the first interview question: “Why is Medicine important to you?” We defined an unambiguous response as one which we could categorize as being either self-focused or other-focused. Self-focused responses focused primarily on the resident (e.g., “I enjoy the challenge,” “I like science”). Other-focused responses focused primarily on others (e.g., “I enjoy helping patients,” “I want to help my community”).^11^ By focusing on the first unambiguous answer we sought to minimize biases in responses resulting from the social nature of the interview (e.g., characteristics of the interviewer and interviewee).^12^

Two investigators not present at the interviews and blinded to any resident identifying information and outcomes used the interview text summaries alone to categorize each resident’s responses as either self-focused or other-focused. If the first response was ambiguous, investigators relied upon the second clarifying response. There were no instances in which investigators required more than the initial two responses to categorize a resident’s focus. The PD resolved all discordant categorizations.

Additional data recorded by the PD after each interview included resident age, sex, and number of previous years as a physician. Other data included responses to questions regarding whether religion or politics played an important role in the resident’s life (binary variables) and geographic region of upbringing.

The primary outcome was the occurrence of any professionalism remedial actions during residency compared between residents with self-focused versus other-focused responses. We defined remediation as any required actions deviating from the standard curriculum: not all actions were adverse. Examples include individual study plans (e.g., reading professionalism literature, preparing professional development self-reflection essays),^13^ written counseling statements, residency probation, and termination. We did not collect data on specific remediation actions taken for each resident in order to maintain resident confidentiality. The decision to start remedial actions rested with a residency committee to which none of the authors belonged. As PD, the lead author was responsible for providing this committee detailed information regarding resident performance. Secondary outcomes included non-professionalism remedial actions (e.g., academic) and residency graduation. We did not collect data on repeat remedial actions. The time horizon over which we measured the primary outcome was the entirety of training for each resident.

We compared characteristics between residents participating in the interviews vs. those not participating to assess for selection bias. We calculated a kappa coefficient to quantify interrater reliability between the two blinded investigators categorizing resident responses to the first interview question as either self-focused or other-focused. We compared all variables and outcomes between residents with self- versus other-focused responses using independent samples Student’s t-tests for continuous variables and Fisher’s exact test or chi-squared test for categorical variables. We calculated the odds ratios (ORs) of professionalism remedial actions based upon independent variables using a logistic regression model to control for confounders. We used SPSS (Version 22, IBM, Armonk, NY) for all statistical analyses.

## RESULTS

Of 114 physicians starting residency during the study, 106 (93.0%) completed the residency entrance interview. The eight remaining residents were unable to participate due to scheduling conflicts on the interview days. The mean age of participants was 31.1 years and 22.6% of participants were female. The proportion of residents undergoing professionalism remedial actions was 15.1%. Examples of actions triggering remediation included absence from assigned shifts, ignoring staff directions, arguments with staff, and negative interactions with patients.

Participant characteristics and outcomes were similar between the 106 participants and eight residents who did not participate. No significant differences existed for any variables between these two groups. Two of the eight residents who did not participate in the interview experienced professionalism remedial actions.

In response to the question “Why is Medicine important to you?,” 50 (47.2%) expressed self-focused answers and 56 (52.8%) expressed other-focused answers based upon categorization by the two blinded investigators (kappa coefficient 0.85). Regarding the primary outcome, a higher proportion of residents expressing self- vs. other-focused answers experienced professionalism remedial actions (26.0% versus 5.4%, p<0.01). A self-focused response was the only subject characteristic significantly associated with the occurrence of professionalism remedial actions during residency in the logistic regression model controlling for potential confounders: OR 8.9 (95% confidence interval 1.8–45.6, Table 2).

## DISCUSSION

This study found an association between self-focused responses to the question “Why is Medicine important to you?” and subsequent resident professionalism-related remedial actions. As described in the psychology literature, self-focus is the conscious direction of attention to one’s self whereas other-focus is the conscious direction of attention to others.^11^,^14^ One potential explanation for our finding is the concept of self-complexity, or the “interrelatedness of various aspects of the person’s conception of self.”^15^ Complex selves “subsume a multiplicity of relationships, traits, goals, and commitments.”^16^ Other-focused responses may reflect residents’ views of themselves as embedded in networks of various roles and responsibilities to different groups of people (such as patients, nurses, and the residents’ own families and friends). Perhaps residents’ self-focused responses reflect a less complex self-concept. People with more complex self-concept may be more resilient to challenges and failures because not every aspect of the self is threatened by setbacks commonly encountered during residency.^15^

Our results contribute to a growing literature regarding physician characteristics and behaviors that correlate with unprofessional behavior. Papadakis et al. importantly highlighted an association between documented unprofessional behavior while in medical school or training and subsequent disciplinary action by medical boards.^2^–^4^ Our investigation expands upon this work by elucidating a resident characteristic that may be identifiable before residents have begun to manifest unprofessional behavior.

## LIMITATIONS

This study has several limitations. First, it reflects retrospective examination of prospectively collect QI data and so our findings are strictly preliminary and we believe should not lead to or impact remedial actions. To the extent that our data compel educators to be more aggressive in initiating remedial actions among self-focused residents, the association identified in our study may become a self-fulfilling prophecy with the potential to inhibit rather than enhance resident professional development. We believe use of our standardized interview tool to select against residency applicants by trying to predict “problem residents” would be similarly ill-advised.^17^ Indeed, while the expression of self-focus to our interview question had high sensitivity in predicting subsequent unprofessionalism, it had very low specificity.

A second limitation is small sample size from a single military EM residency program. Thus, it is difficult to speak to the generalizability of our results to civilian training programs and programs in other specialties. Also our small sample size limited the power of our regression analysis, and it is possible that other important correlates with unprofessional behavior exist.

A third limitation is that the categorization of the independent variable of each resident’s reason for valuing a career in Medicine as being primarily other- or self-focused was arguably subjective. We based these categorizations upon the free-text written records of the PD, which were finalized in consultation with each resident at interview completion to ensure accuracy. It is possible that the interview discussion following the resident’s response to the first question may have influenced this text record. Further, other interviewers may have either solicited different resident responses or recorded the same verbal responses differently.

Another limitation is our primary outcome measure of professionalism remedial action. Measuring professionalism is complex and the literature describes myriad methods for making this measurement.^18^–^20^ Indeed, research suggests poor inter-rater reliability among academic faculty in determining what constitutes unprofessional behavior.^21^ In our training program, the decision to impose a professionalism remedial action was the collaborative decision of a Clinical Competency Committee (CCC).^22^ The CCC comprised multiple faculty members, none of whom were involved with this study. The educational literature supports the use of such committees as an effective mechanism for identifying unprofessional behavior.^23^ We believe the collaborative nature of these decisions makes this outcome measure more reliable. Nevertheless, it is impossible to say whether the same resident actions would have led to similar remedial outcomes at other programs. Our decision to not collect data regarding each resident’s specific remedial action plan to protect resident confidentiality further complicates efforts to extrapolate our experience to other settings.

Future work might study the impact on resident professionalism of other factors not explored in this study including social upbringing, childhood education, hospital environment, and the “hidden curriculum.”^24^,^25^ It would also be interesting to administer our interview tool in different contexts. In particular, future studies could examine whether simply educating residents about the association we identified in this study to stimulate their own introspection into the motivations underlying their career choices might be effective in decreasing instances of unprofessional behavior.

## CONCLUSION

Self-focused professional values as expressed during our standardized interview correlated with professionalism remedial actions during residency. These results may aid medical educators in identifying residents who may benefit from additional attention to professional development.

## Supplementary Information



## Figures and Tables

**Table 1:** Characteristics of all interviewed emergency medicine residents starting training during 2006–2013, stratified by self-focused versus other-focused interview responses (n=106).

Characteristics	All residents, n=106	Self-focused, n=50	Other-focused, n=56	p*
Mean age, years	31.1	30.3	31.8	0.12†
Female sex, %	22.6	28.0	17.9	0.25‡
Mean pre-residency time as physician, years	0	0.4	1.0	0.09§
Geographical home of record, %				
New England (CT, ME, MA, NH, RI, VT)	5.7	8.0	3.6	
Mid-Atlantic (NJ, NY, PA)	14.2	14.0	14.3	
East north central (IL, IN, MI, OH, WI)	16.0	12.0	19.6	
West north central (IA, KS, MN, MO, NE, ND, SD)	7.5	4.0	10.7	
South-Atlantic (DA, FL, GA, MD, NC, SC, VA, DC, WV)	15.1	20.0	10.7	
East south central (AL, KY, MS, TN)	2.8	6.0	0	
West south central (AR, LA, OK, TX)	8.5	10.0	7.1	
Mountain (AZ, CO, ID, MT, NV, NM, UT, WY)	18.9	16.0	21.4	
Pacific (AK, CA, HI, OR, WA)	11.3	10.0	12.5	0.33**
Religion as significant life influence (%)	51.9	38.0	64.3	0.01‡
Politics as significant life influence (%)	29.2	40.0	19.6	0.03‡
Graduated residency (%)	95.3	98.0	92.9	0.37‡
Non-professional remedial action (%)	20.8	20.0	21.4	1.00‡
Professional remedial action (%)	15.1	26.0	5.4	<0.01‡

*CT*, Connecticut; *ME*, Maine; *MA*, Massachusetts; *NH*, New Hampshire; *RI*, Rhode Island; *VT*, Vermont; *NJ*, New Jersey; *NY*, New York; *PA*, Pennsylvania; *IL*, Illinois; *IN*, Indiana; *MI*, Michigan; *OH*, Ohio; *WI*, Wisconsin; *IA*, Iowa; *KS*, Kansas; *MN*, Minnesota; *MO*, Montana; *NE*, Nebraska; *ND*, North Dakota; *SD*, South Dakota; *DE*, Delaware; *FL*, Florida; *GA*, Georgia; *MD*, Maryland; *NC*, North Carolina; *SC*, South Carolina; *VA*, Virginia; *DC*, District of Columbia; *WV*, West Virginia; *AL*, Alabama; *KY*, Kentucky; *MS*, Mississippi; *TN*, Tennessee; *AR*, Arkansas; *LA*, Louisiana; *OK*, Oklahoma; *TX*, Texas; *AZ*, Arizona; *CO*, Colorado; *ID*, Idaho; *MT*, Montana; *NV*, Nevada; *NM*, New Mexico; *UT*, Utah; *WY*, Wyoming; *AK*, Alaska; *CA*, California; *HI*, Hawaii; *OR*, Oregon; *WA*, Washington.

* Values reflect comparisons of characteristics between residents expressing self-focused values versus those expressing other focused values.

† Two-tailed independent samples Student’s t-test (equivalent variances by Levene’s test).

‡ Fisher’s exact test (2-sided).

§ Two-tailed independent samples Student’s t-test (non-equivalent variances by Levene’s test).

** Chi-squared test.

**Table 2:** Logistic regression model measuring associations between resident characteristics and the occurrence of professionalism remedial actions (n=106).

		95% Confidence interval	
		
Variables*	Odds ratio	Lower	Upper
Age	1.1	0.9	1.3
Female sex	0.5	0.1	3.1
Pre-residency time as physician	0.3	0.0	1.5
Pre-residency time as active duty military service member	1.6	0.2	11.5
Religion as significant life influence	2.2	0.6	8.7
Politics as significant life influence	1.3	0.3	5.1
Self-focused interview question response	8.9	1.8	45.6

* All variables are binary except for age for which the odds ratio reflects the association with professionalism remedial action with each year increase.
