# Supplementary material for: Self vs. Other Focus: Predicting Professionalism Remediation of Emergency Medicine Residents
Source: West J Emerg Med. 2017 Dec 14;19(1):35–40. doi: 10.5811/westjem.2017.11.35242 (PMC5785199; doi:10.5811/westjem.2017.11.35242)
Supplement: Supplementary file 1 [file wjem-19-35-s001.docx]

**APPENDIX TABLE.** Examples of additional questions asked during standardized interview (2006-2013).

|  |
| --- |
| **Questions** |
| What have been your experiences as a healthcare provider prior to residency? |
| What have been your military assignments prior to residency? |
| Where is your childhood home? |
| Why did you choose Emergency Medicine for your specialty? |
| Is religion a significant influence in your life? |
| Are politics a significant influence in your life? |
